# Sex-specific transcriptomic and epitranscriptomic signatures of PTSD-like fear acquisition

**DOI:** 10.1016/j.isci.2022.104861

**Published:** 2022-08-02

**Authors:** Andre L.M. Reis, Jillian M. Hammond, Igor Stevanovski, Jonathon C. Arnold, Iain S. McGregor, Ira W. Deveson, Anand Gururajan

**Affiliations:** 1 Genomics Pillar, Garvan Institute of Medical Research, Sydney, NSW 2010, Australia; 2 Centre for Population Genomics, Garvan Institute of Medical Research and Murdoch Children’s Research Institute, Sydney, Australia; 3 School of Clinical Medicine, Faculty of Medicine and Health, UNSW Sydney, Sydney, NSW 2010, Australia; 4 Faculty of Medicine and Health, Sydney Pharmacy School, Discipline of Pharmacology, The University of Sydney, Sydney, NSW 2050, Australia; 5 Brain & Mind Centre, The University of Sydney, Sydney, NSW 2050, Australia; 6 The Lambert Initiative for Cannabinoid Therapeutics, Australia; 7 School of Psychology, Faculty of Science, The University of Sydney, Sydney, NSW 2050, Australia

**Keywords:** Molecular biology, Molecular mechanism of behavior, Behavioral neuroscience, Omics, Transcriptomics

## Abstract

Our understanding of the molecular pathology of posttraumatic stress disorder (PTSD) is evolving due to advances in sequencing technologies. With the recent emergence of Oxford Nanopore direct RNA-seq (dRNA-seq), it is now also possible to interrogate diverse RNA modifications, collectively known as the “epitranscriptome.”. Here, we present our analyses of the male and female mouse amygdala transcriptome and epitranscriptome, obtained using parallel Illumina RNA-seq and Oxford Nanopore dRNA-seq, associated with the acquisition of PTSD-like fear induced by Pavlovian cued-fear conditioning. We report significant sex-specific differences in the amygdala transcriptional response during fear acquisition and a range of shared and dimorphic epitranscriptomic signatures. Differential RNA modifications are enriched among mRNA transcripts associated with neurotransmitter regulation and mitochondrial function, many of which have been previously implicated in PTSD. Very few differentially modified transcripts are also differentially expressed, suggesting an influential, expression-independent role for epitranscriptional regulation in PTSD-like fear acquisition.

## Highlights

• PTSD-like trauma has sexually dimorphic effects on the amygdala transcriptome
• Most RNA modifications identified adhere to the known patterns associated with m6A
• There was enrichment of RNA modifications in neurological/PTSD-related genes
• There was little overlap between transcriptomic and epitranscriptomic signatures

## Graphical abstract

**Figure:**
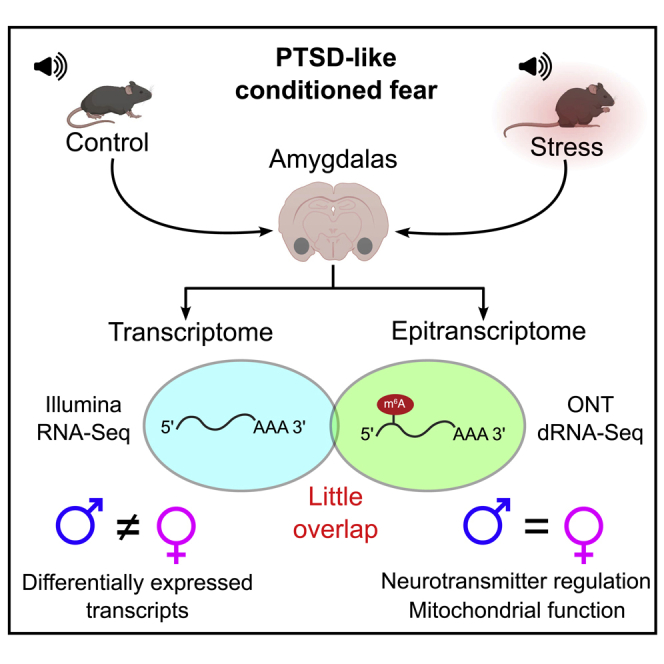

## Introduction

Posttraumatic stress disorder (PTSD) is rooted in the acquisition of memories associated with specific traumatic events (Maren and Holmes, 2016). Evidence from clinical imaging research and studies using animal models of aspects of PTSD suggests that PTSD pathophysiology manifests from a dysregulation of the amygdala in response to trauma-related or emotional cues (Verbitsky et al., 2020; Del Casale et al., 2022; Johansen et al., 2011; Leite et al., 2022; Gonda et al., 2022; Yehuda et al., 2015).

Accumulating evidence indicates that epigenetic (dys)regulation could play a pivotal role in the pathogenesis of PTSD (Zannas et al., 2015; Howie et al., 2019). However, over the last decade, new research has shifted attention from the epigenome to the role of RNA modifications, collectively known as the epitranscriptome, in modulating brain function and behavior (Zhao et al., 2017; Madugalle et al., 2020; Livneh et al., 2020; Jonkhout et al., 2017; Murakami and Jaffrey, 2022). These modifications may impact RNA stability, localization, and splicing and regulate translation in a dynamic and reversible manner. As such, they have the potential to rapidly fine-tune responses to external stimuli and may underpin aspects of fear learning and memory (Widagdo et al., 2016; Walters et al., 2017; Engel et al., 2018). However, relatively little is currently known about the epitranscriptome, and its regulatory roles in the brain, due to technological limitations.

The recent emergence of nanopore sequencing technology enables direct sequencing of full-length native RNA molecules, circumventing the need for reverse transcription or amplification (Deamer et al., 2016). As an RNA molecule passes through a protein pore embedded in a synthetic membrane, there is a disruption in the flow of ions across the membrane, in a predictable manner depending on the composition of the bases within the pore at any given moment. This technology enables not only sequencing of full-length RNA molecules but also the detection of RNA modifications, which induce discernible shifts in current intensity. Therefore, direct RNA nanopore sequencing may be an ideal tool for comprehensive profiling of the epitranscriptome in different biological contexts (Ameur et al., 2019).

**Figure 1:**
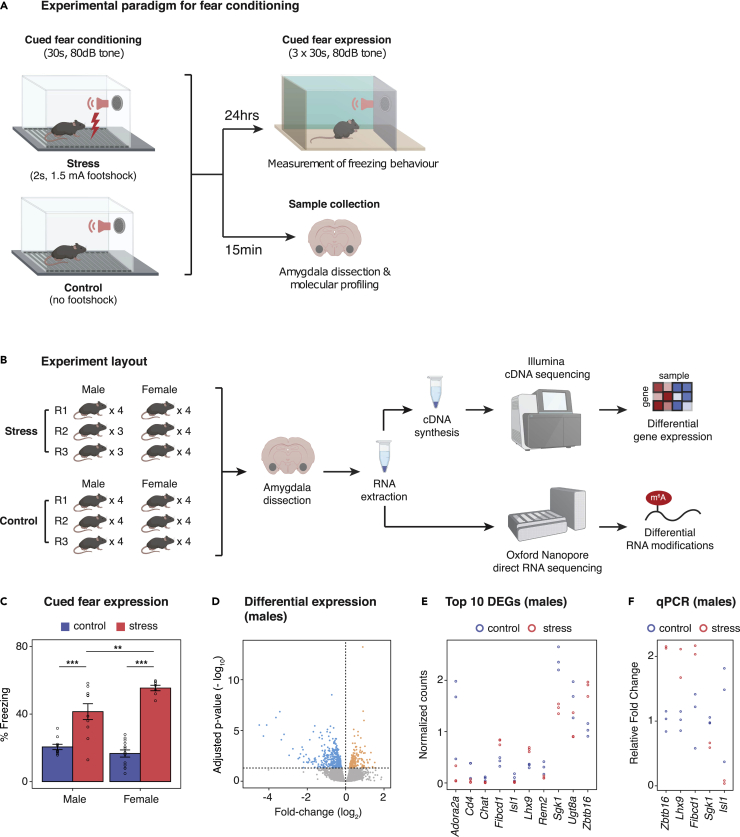
Sex-specific transcriptional profiles are associated with the acquisition of PTSD-like fear memories in the amygdala (A) Adult male and female mice underwent cued-fear conditioning (or the control condition) after which they were tested for cued-fear expression 24 h later; another group was sacrificed within 15 min of fear conditioning to obtain the amygdala. (B) RNA from the amygdala of adult male and female mice from both experimental groups (control or cued-fear) was subsequently pooled (3–4 individuals per pool or 12 individuals per condition) for indirect (Illumina) and direct (Oxford Nanopore) sequencing. (C) Face validity of the cued fear conditioning paradigm was verified in male and female mice, which showed strong expression of conditioned fear responses. ANOVA, ∗∗∗p < 0.001 relative to non-fear-conditioned controls, ∗∗p < 0.01 for male- versus female-conditioned mice. Data are represented as mean ± SEM (n = 7–10/group). (D) Differential gene expression in the amygdala of male mice; blue points represent significantly downregulated genes; yellow points represent significantly upregulated genes, and gray points represent nonsignificant genes. Thresholds for DESeq2: |FC|>20% and FDR <0.05. (E) Top 10 differentially expressed genes in male-conditioned mice. (F) qPCR validation for selected differentially expressed genes in males.

Here we employed a well-validated mouse model of aspects of PTSD to investigate transcriptional changes in the amygdala during the acquisition of conditioned fear response (Figure 1A) (Li et al., 2005; Siegmund and Wotjak, 2007; Kao et al., 2016; Assareh et al., 2020; Brzozowska et al., 2017; Dedic et al., 2018). Because we were interested in capturing the earliest molecular changes, we collected tissue for RNA extraction just 15 min after exposure to the fear conditioning cue, consistent with previous studies (Rosen et al., 1998; Huff et al., 2006). We leveraged parallel short-read RNA-seq (Illumina NextSeq) to profile gene expression and long-read Oxford Nanopore Technologies (ONT) direct RNA-seq (ONT PromethION) to build a comprehensive catalog of RNA modifications associated with conditioned fear response (Figure 1B).

## Results

Our PTSD mouse model was developed using a modified auditory, cued-fear conditioning protocol developed by Siegmund and Wotjak (2007) and has been validated for its face, construct, and predictive validities in male mice (Kao et al., 2016; Assareh et al., 2020; Dedic et al., 2018; Smith et al., 2019; Brzozowska et al., 2017; Li et al., 2005). Accordingly, for our first experiment, we investigated if cued-fear memory was also observable in adult female mice 24 h after fear conditioning. We observed a significant increase in freezing behavior in both male and female conditioned (CS tone, shocked) mice compared with nonconditioned (CS tone, nonshocked) controls, with females freezing significantly more than males (Figure 1C). Our findings were consistent with one other study that also reported greater cued-fear expression in females than males, suggestive of sex differences in discriminative ability to the cue (ter Horst et al., 2012).

These findings led us to investigate whether this significant sex difference in expression of PTSD-like fear memory could be associated with differences in transcriptional and epitranscriptional processes associated with the initial acquisition of the fear memory. To address this, a separate group of mice was sacrificed within 15 min of the fear conditioning procedure, capturing the earliest molecular signatures of fear acquisition (Figure 1A). mRNA was extracted and isolated from the amygdala of each individual, pooled in groups of 3–4 individuals per condition, and analyzed by short-read RNA-seq to assess differential gene expression responses to the PTSD-like stressor (Figure 1B). We obtained on average 37.9 million read pairs per sample (SD = 7.23 x 10^6^; Table S1). In each library, most of the reads were successfully mapped to the reference genome (84.72% ± 0.56) and subsequently assigned to an annotated gene (65.83% ± 1.03; Table S2).

We first determined differences in gene expression in male and female mice exposed to the CS tone only (i.e., the control group). These mice did experience a novel environment, and their gene expression profile should therefore be considered not as “baseline” but as mildly stressed. There were 129 differentially expressed genes in the amygdala of females compared with males, including a number of genes previously identified as sexually dimorphic (Figures S1A and S1B) (Yang et al., 2006; Deschepper, 2020). Importantly, 5.1% of dimorphic expression changes observed within this female versus male control comparison were also observed in response to fear conditioning (Figures S1C, Tables S3, and S4).

We identified 190 upregulated and 401 downregulated genes in conditioned male mice (fold-change > 0.2 and FDR <0.05; Figures 1D and Table S5). We compared our list of differentially expressed genes in males with those found in another study that examined the amygdala transcriptome 2 h following auditory fear conditioning (Lori et al., 2019). There were 53 out of the 591 genes that overlapped; despite differences in methodology (e.g., foot-shock current 0.6 mA versus 1.5 mA), we speculate that changes in the expression of these genes may reflect a general, temporally independent response to the fear conditioning paradigm. In males, the expression of the top 10 differentially expressed genes (DEGs) included Zinc finger and BTB domain containing 16 (*Zbtb16*), LIM homeobox protein 9 (*Lhx9*), fibrinogen C domain containing 1 (*Fibcd1*), serum/glucocorticoid regulated kinase 1 (*Sgk1*), and Isl1 transcription factor, LIM/homeodomain (*Isl1*), all of which were qPCR validated using the same samples that were sequenced (Figures 1E and 1F).

*Zbtb16* was found to be significantly upregulated in conditioned male mice and is a transcriptional regulator involved in a myriad of processes including those linked to neurodevelopment (Lin et al., 2019; Gaber et al., 2013). *Zbtb16* knockout mice show several behavioral impairments relevant to neurodevelopmental disorders such as autism spectrum disorder and schizophrenia. In addition to impaired cortical morphology, these mice also show dysregulation in genes associated with myelination and neurogenesis (Usui et al., 2021). Functional expression of this gene in the adult hypothalamus has been recently linked to regulation of metabolism (Cheng et al., 2020). *Lhx9* and *Isl1* are transcription factors that also have roles in neurodevelopment (Garcia-Calero and Puelles, 2021; Zhuang et al., 2013). *Fibcd1* belongs to a class of proteins known as fibrinogen-related domains (FreD) with multiple functions that include innate immunity and are expressed in glial cells in the human brain (von Huth et al., 2018). To our knowledge, the functional expression of these four genes in the adult amygdala and in response to PTSD paradigms such as the one used in our study has heretofore not been characterized. They therefore represent interesting targets for future investigations.

*Sgk1* was downregulated in expression. It is involved in several functions, which include regulating ion channel activity, interfering with transcription, and neuronal excitability (Lang et al., 2006). In the amygdala, an earlier microarray study reported an increase in *Sgk1* expression following cued-fear conditioning, which contrasts with our findings; however, these experiments were carried out using a different conditioning protocol, and analysis of amygdala RNA was at later time points (Andero et al., 2014). It is worth noting that *Sgk1* is also expressed in other brain regions including the hippocampus, where it has a role in contextual fear memory formation (Lee et al., 2007) and in the prefrontal cortex (PFC). A downregulation in expression has been reported in postmortem tissue analyses of PFC tissue from patients who had suffered from PTSD and is associated with increased expression of contextual fear memory in rats (Licznerski et al., 2015).

Independent Gene Set Enrichment Analysis (GSEA) for conditioned male mice indicated a significant overrepresentation of genes implicated in synaptic activity (Table S6), a process that is well known to be implicated in the acquisition of memories (Takeuchi et al., 2013; Silva, 2003).

Epidemiological evidence suggests that females are at higher risk (2:1) of developing PTSD following trauma with sex-specific central and peripheral patho-transcriptional signatures (Breen et al., 2018; Christiansen and Berke, 2020; Girgenti et al., 2021). However, to our knowledge, few PTSD-stress paradigms, including the one we have employed here, have been applied in females (Verbitsky et al., 2020). To our surprise, despite the increased expression of cued-fear memory, fear conditioning induced a modest transcriptional response in conditioned female mice. We observed a significant decrease in expression for only a single gene, *Rps27A*; however, this was not validated using qPCR. There remains debate as to whether the amygdala is a sexually dimorphic structure (Marwha et al., 2017; Wolf et al., 2021; Chatzinakos et al., 2021; Montalvo-Ortiz et al., 2022). Evidence suggests there are intrinsic differences linked to sex hormones that may explain our sex-specific observations in the formation and expression of emotional memories (Blume et al., 2017; Morris et al., 2008; Cooke and Woolley, 2005). Overall, our gene expression data suggest significant distinctions exist during the early transcriptional events of fear acquisition in males and females, warranting much further investigation.

Recent evidence suggests that epitranscriptomic modifications, such as N6-methyladenosine (m6A), may have widespread regulatory roles in the brain (Jonkhout et al., 2017; Livneh et al., 2020; Madugalle et al., 2020; Zhao et al., 2017). Oxford Nanopore Technology (ONT) dRNA sequencing has the potential to enable comprehensive profiling of the diverse array of RNA base modifications that together comprise the “epitranscriptome” (Oikonomopoulos et al., 2020; Wang et al., 2021b). However, this is a nascent field, and analytical best practices are yet to be established (Amarasinghe et al., 2020).

To explore epitranscriptome dynamics during fear acquisition, we performed dRNA sequencing on the same amygdala RNA samples as described above. Each sample was sequenced on a single PromethION flow cell, yielding a median of 3.9 million QC-passed reads per sample, each of which represents a native mRNA transcript (Table S7). This enabled a broad survey of expressed transcripts (average read count >3), with 12,792 out of 32,604 (39.2%%) of protein coding genes reaching an average read count greater than 15, the minimum threshold required for detection of putative RNA modifications (Table S8).

**Figure 2:**
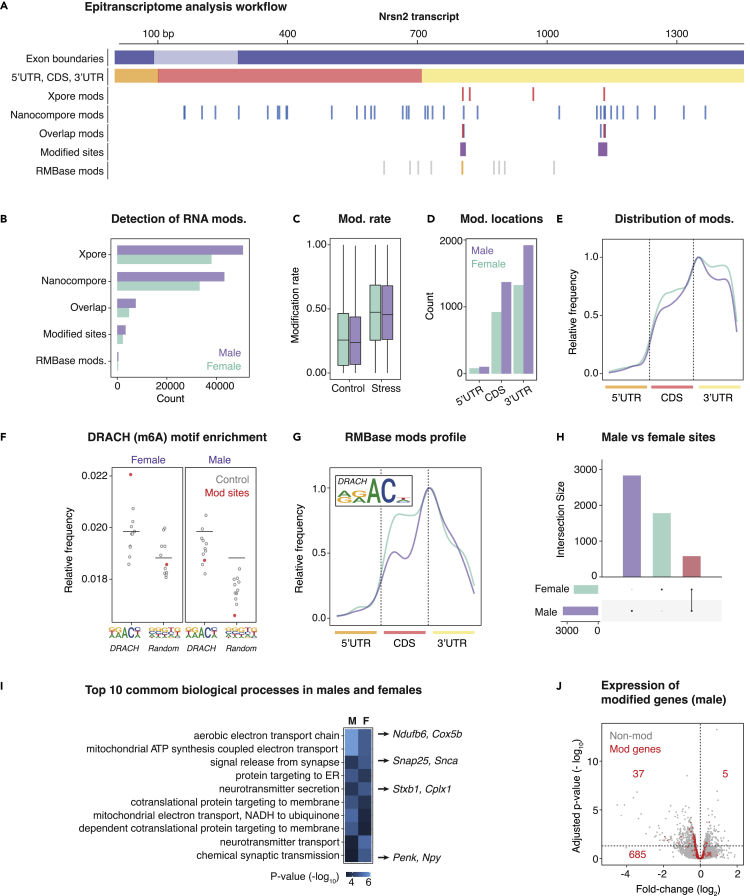
Epitranscriptome signatures of PTSD-like fear acquisition in the amygdala (A) Schematic example using the *Nrsn2* mRNA transcript to illustrate the analytic workflow for profiling RNA modifications in conditioned versus control mice. Briefly, candidate modifications were identified separately by *Xpore* and *Nanocompore*. Only overlapping/adjacent candidates identified by both tools were retained, and multiple candidates within 10 nt were collapsed into high-confidence modified sites. Finally, modified sites were cross-checked against RMBase. (B) The number of candidates and modified sites retained at each step of the workflow just described. (C) The modification rates recorded by *Xpore* for modified sites in stress versus control and male versus female samples. (D) The total number of modified sites identified within different regions (5′UTR, CDS and 3′UTR) of mRNA transcripts. (E) The positional distribution of modified sites, averaged across the whole transcriptome, within the same regions (5′UTR, CDS and 3′UTR). (F) The relative frequency of canonical DRACH motif Kmers versus random k-mers observed within modified sites (blue), as opposed to random sites in matched transcript regions. (G) The positional distribution and k-mer frequency profile of modified sites that also overlapped RMBase modifications, with both showing characteristic features of m6A. (H) The number of modified sites identified that were sex specific or shared between males and females. (I) The top ten common GO terms enriched among differentially modified genes in males and females. Example genes underlying each term are highlighted. (J) Volcano plot showing gene expression fold-changes observed between stress versus control, with genes that were identified as differentially modified highlighted in red. Minimal overlap between differentially expressed and differentially modified genes is apparent.

An overview of our analysis workflow used to profile RNA modifications is shown in Figure 2A, exemplified using the transcript for Neurensin-2 (*Nrsn2*), a known modulator of emotional behavior and putative biomarker for PTSD (Umschweif et al., 2021; Glatt et al., 2013). Briefly, we used a combination of two recently developed software packages, *Xpore* (Pratanwanich et al., 2021) and *Nanocompore* (Leger et al., 2019), to detect differential RNA modifications in the comparison of conditioned versus controls samples. *Xpore* identified 50,681 candidate modification sites across 9,902 transcripts for males and 37,998 candidates across 8,979 transcripts for females (Figure 2B). *Nanocompore* identified 43,172 candidate modification sites across 1,547 transcripts for males and 33,216 candidates across 1,482 genes for females (Figure 2B). The tools use alternative metrics and statistical approaches to identify modified k-mers, and concordant results provide the best evidence of modified sites. To maximize stringency, we retained only those candidates who were identified by both *Nanocompore* and *Xpore* and collapsed any candidates within 10 nt into a single modification site (Figure 2A). For males, this approach identified 7,487 candidate modifications between conditioned versus control conditions that were collapsed into 3,397 unique differentially modified sites (Figure 2B). In females, 4,808 candidates were collapsed into 2,331 unique differentially modified sites (Figure 2B). These high confidence sites showed mean ∼1.7-fold higher modification rates, as measured by displacement of the current signal, under the stress condition in both males (stress = 0.48, control = 0.27) and females (stress = 0.49, control = 0.29; Figure 2C).

Most high-confidence modified sites were found within 3′ untranslated regions (UTR) of mRNA transcripts, with an enrichment close to the boundary with the coding sequence (CDS) observed in both males and females (Figures 2D and 2E). A smaller proportion of modified sites were found within 5′UTRs, which are generally shorter than 3′UTRs, but 5′UTRs exhibited the highest density of sites per nucleotide compared with the 3′UTR and CDS in both sexes (Figures S1D and S1E). These observations are consistent with positional distributions previously reported for m6A and other known RNA modifications (Meyer et al., 2012, 2015; Dominissini et al., 2012).

Although *Nanocompore* and *Xpore* can identify differentially modified bases, neither tool determines the specific type of RNA modification at a given site. To further characterize and validate our modification sites, we assessed the enrichment of sequence k-mers consistent with the canonical m6A DRACH motif (Wang et al., 2021a). We found an enrichment of DRACH motif k-mers within high-confidence modification sites, compared with 10 sets of random nonmodified sites, and no enrichment for k-mers of a randomly selected motif (Figure 2F). We also intersected our set of high-confidence modification sites with RMBase, a database of known RNA modifications (Xuan et al., 2017). We found 506 annotated RMBase modifications overlapping differentially modified sites in males and 421 in females, with almost all of them identified as m6A (Figure 2B). We retrieved the consensus sequence around these m6A sites and consistently observed the DRACH motif for both sexes (Figure 2G). Concordance with previously annotated RNA modifications and adherence to known patterns of transcript position and sequence context confirms the reliability of our catalog of differentially modified sites during fear acquisition.

Next, we functionally characterized genes and transcripts identified as being differentially modified. Out of 3,397 and 2,331 modified sites detected in males and females (Tables S9 and S10), respectively, we found 580 common modifications in 186 genes (average = 3.11 sites/gene; Figure 2G; Figure S1F), suggesting a degree of both shared and sexually dimorphic epitranscriptome dynamics. GSEA for males and females indicated enrichment of equivalent and/or related gene ontology terms (Figure 2I). Within the top 20 most significantly enriched terms, 60% were found in both males and females (Tables S11, S12, and S13).

Mitochondrial function and neurotransmitter production/secretion were the predominant functional categories identified by GSEA (Figure 2I). The former is particularly interesting in the context of evidence implicating mitochondrial function in the stress response (Daniels et al., 2020; Manoli et al., 2007; Picard et al., 2018) and mitochondrial dysfunction in PTSD pathology (Zhang et al., 2015; Flaquer et al., 2015). Mitochondrial metabolic processes are components of the cell danger response (CDR) that is activated by stressors. Negative feedback mechanisms exist to “switch off” the CDR once the stress has passed but if they fail, this may contribute to oxidative stress and inflammation (Naviaux, 2014).

Among the top differentially modified gene candidates were multiple synaptic genes previously implicated in PTSD, including neuropeptide Y (*Npy*) (Sah and Geracioti, 2013). Evidence suggests the *Npy* system promotes resilience-to-stress in rodents and reduced NPY in humans is associated with PTSD (Sah and Geracioti, 2013). We observed differentially modified sites in the 3′UTR and CDS regions of *Npy* in males and females. In each case, the modification frequency was upregulated during fear conditioning, whereas expression of *Npy* mRNA was not found to be upregulated, suggesting transcription-independent regulation of *Npy* by RNA modifications may occur during fear conditioning. The lack of concordance between differential modification and expression in *Npy* was similarly observed across the transcriptome, with just 150/3,397 (4.41%) of high-confidence modifications being harbored within 42 differentially expressed genes (Figure 2J; Table S14).

## Discussion

Gaining an insight into the molecular mechanisms that are triggered immediately following trauma is important to comprehend how they are implicated in the neuropathological cascades that culminate in the onset of PTSD. Our study provides a framework for exploring acquisition-induced transcriptomic and epitranscriptomic events underpinning PTSD-like phenotypes in mice. Our results show that any effect of RNA modifications does not result in obvious gene expression changes at an early stage of fear conditioning. Therefore, it is possible that RNA modifications do not have any role at all in that process, but their enrichment in neurological/PTSD-related genes is an interesting finding that warrants further investigation. There is broad scope for future work, but one major issue must be addressed: how does one interrogate the functional roles of RNA modifications (Schaefer et al., 2017).

For some modifications such as m6A, the majority of known m6A sites are unmethylated at baseline (He and He, 2021). The functional relevance of constitutive versus regulated m6A sites is unknown but such sub-stoichiometric levels would indicate a large margin in which to regulate RNA metabolism and gene expression. Furthermore, although the life cycle of modifications is thought to be transient, this may vary from transcript to transcript. Our data are also consistent with recent work showing that RNA modifications can form clusters on the same transcript (Zhang et al., 2019), exerting cumulative or combinatorial effects on RNA metabolism. One low-resolution approach to providing answers is by manipulating the expression and activity of epitranscriptomic machinery enzymes—“writers,” “erasers,” and “readers.” This has been used by numerous studies in the context of learning, memory, and the stress response (Walters et al., 2017; Widagdo et al., 2016; Engel et al., 2018). There have also been several pharmacological compounds developed to inhibit or activate these enzymes (Huang et al., 2019; Selberg et al., 2021). One higher resolution strategy involves the use of CRISPR-Cas-inspired RNA-targeting (CIRT) system (Rauch et al., 2018, 2019) but this is yet to validated *in vivo*.

In conclusion, our study opens a new and exciting frontier in molecular psychiatry research that has potential to reshape our understanding of stress-induced psychiatric disorders. RNA modifications could be among the earliest molecular events associated with fear conditioning, but more studies are needed at additional time points, using proteomics and ribosome profiling among other techniques to disentangle the many possible (and nonmutually exclusive) impacts of specific m6A sites to the pathophysiology of PTSD (Engel et al., 2018). RNA-based therapies have found success in treatment of diseases from cancer to COVID19 (Damase et al., 2021). Perhaps, there is scope to treat psychiatric disorders such as PTSD in a similar manner.

### Limitations of the study

Each sample analyzed in our study was a pool of 3–4 independent individuals, meaning a total of 12 different individuals per condition were represented. Sequencing each individual as a separate biological replicate rather than pooling would increase the statistical power of our study; however, due to the high cost and large RNA input quantity required for direct-RNA sequencing, adding more samples to our study was unfeasible. Sample multiplexing is not supported for direct-RNA sequencing, so each sample must be sequenced on a separate ONT PromethION flow cell, resulting in prohibitively expensive costs for large experimental designs. Furthermore, although we were focused on capturing the earliest transcriptional changes associated with fear conditioning, additional time points will illuminate the temporal relationships between transcript abundance and transcript modification to the pathophysiology of PTSD. In addition, the use of proteomics techniques, such as ribosome profiling, will enable the understanding of the real impact of transcriptomic and epitranscriptomic changes to the acquisition of traumatic memories at the molecular level.

## STAR★Methods

### Key resources table

**Table:** 

REAGENT or RESOURCE	SOURCE	IDENTIFIER
**Critical commercial assays**		

QIAGEN RNeasy Mini Kit (250)	Qiagen	74106
Dynabeads™ mRNA Purification Kit	ThermoFisher Scientific	61006
Direct RNA sequencing kit	Oxford Nanopore Technologies	SQK-RNA002
Flow Cell Priming Kit	Oxford Nanopore Technologies	EXP-FLP002
PromethION Flow Cell	Oxford Nanopore Technologies	FLO-PRO002
Ethanol absolute for analysis	Merck	1009831000
Nuclease-free water	Qiagen	129114
SuperScript III Reverse Transcriptase	ThermoFisher Scientific	18080044
10 mM dNTP solution	NEB	N0447
NEBNext® Quick Ligation Reaction Buffer	NEB	B6058
T4 DNA Ligase 2M U/ml	NEB	M0202
Agencourt RNAClean XP beads	Beckman Coulter	A63987
Qubit RNA HS Assay Kit	ThermoFisher Scientific	Q32852
Qubit dsDNA HS Assay Kit	ThermoFisher Scientific	Q32851
KAPA Stranded RNA-Seq Library Preparation Kit	Roche	KR0934
SeqCap Adapter Kit A	Roche	07141530001

**Deposited data**		

Raw data	This paper	GEO:
Mouse reference genome (mm10)	Genome Reference Consortium	https://www.ncbi.nlm.nih.gov/assembly/GCF_000001635.20/
Mouse gene annotation (M25)	Gencode	https://www.gencodegenes.org/mouse/release_M25.html
Raw sequence reads	SRA	PRJNA779821

**Experimental models: Organisms/strains**		

Mus musculus C57BL/6JArc	Australian Resource Centre	Cat No.: 000664

**Software and algorithms**		

R		Version 3.6.3
R Studio	https://www.rstudio.com/products/rstudio	Version 1.2.5033
Minimap2	https://github.com/lh3/minimap2	Version 2.17-r941
Nanocompore	https://github.com/tleonardi/nanocompore	v1.0.3
Xpore	https://github.com/GoekeLab/xpore	Version 2.0
F5c	https://github.com/hasindu2008/f5c	Version 0.8
Scripts	Zenodo	https://doi.org/10.5281/zenodo.6782309

### Resource availability

#### Lead contact

Further information and requests for resources and reagents should be directed to and will be fulfilled by the lead contact, Dr Anand Gururajan (anand.gururajan@sydney.edu.au).

#### Materials availability

This study did not generate new unique reagents.

#### Data and code availability

Raw sequencing data has been uploaded to the NCBI SRA (PRJNA779821) and are publicly available as of the date of publication. Accession numbers are listed in the key resources table.
All original code has been deposited at Zenodo and is publicly available as of the date of publication. DOIs are listed in the key resources table.
Any additional information required to re-analyse the data reported in this paper is available from the lead contact upon request.

### Experimental model and subject details

The study used 22 adult male and 24 adult female (10 weeks of age) C57BL/6JArc mice purchased from the Animal Resource Centre, Western Australia. All experiments with mice conformed to the regulatory standards approved by the University of Sydney, Animal Ethics Committee (2018/1425). As per the approved ethics protocol, all animals were initially group-housed for 1 week followed by single housing in individually ventilated cages with environmental enrichment and ad-libitum access to food and water. Cages were in racks in a room maintained in a temperature/humidity-controlled environment (21°C, 55.5%) with reverse-phase lighting (lights ON: 21h00, OFF: 09h00). In our experiments, we included the use of randomly cycling female mice (Shansky and Woolley, 2016).

### Method details

#### Cued fear conditioning and cued fear memory testing

Mice were fear conditioned in a wooden sound-proof chamber, transparent front and rear-facing walls, opaque side walls with a white light and a metal grid floor (Context A). The conditioning session started with a 198 s acclimation period which was followed by a 30 s 80dB tone (9KHz sinewave, conditioned stimulus, CS) that co-terminated with a 2s 1.5mA foot-shock delivered through the metal grid as a constant current (unconditioned stimulus, US). The conditioning session ended with a 60 s after-shock interval. Non-shock control mice were exposed only to the CS tone. The conditioning session from start to finish is just under 5 min. Fear conditioning chambers were cleaned using 70% ethanol spray solution. Within 15 mins of being fear conditioned, one group of stress and control mice were culled, brains extracted and snap frozen. Cued fear memory was tested in another group of stress and control mice 24hrs later in a novel context (Context B: wooden sound-proof chamber, red light, red perspex flooring, peppermint oil scent). Following a 2 min baseline period, all mice were presented with 3 x 30 sec CS with an inter-tone interval of 1 min. The memory retrieval session ended with a 60 s interval after the last tone was presented. Chambers were cleaned using F10 disinfectant spray solutions. Freezing was measured in response to the CS using the CleverSys FreezeScan® video tracking system and software.

#### Tissue collection & RNA extraction

Mice were sacrificed by cervical decapitation within 15 min of being fear conditioned. Brains were extracted, snap-frozen in liquid nitrogen and stored at -80°C. Bilateral punches (0.5 to 1mm) of the amygdala (AMG: central and basolateral amygdala) were made using the Paxinos and Watson Atlas as a guide (Paxinos, 2004; Zapala et al., 2005). Tissue was collected in RNA-free tubes and RNA was extracted using Qiagen RNeasy^TM^ Micro kit (Qiagen, MD, USA) according to the manufacturer’s instructions. RNA concentrations were quantified using a NanoDrop™ One spectrophotometer (Thermofisher Scientific®, MA, USA) and only samples with 260/280 ratios of greater than 1.7 were used for downstream analyses. Samples from 3-4 mice were randomly pooled for subsequent analyses by Illumina NextSeq and Promethion, meaning that a total of 12 different individuals were represented in each condition. mRNA was purified from total RNA pools using the Dynabeads® mRNA Purification Kit (Thermofisher Scientific®, MA, USA) according to the manufacturer’s instructions.

#### Illumina RNA sequencing

Sequin RNA Mix (Hardwick et al., 2016) sequencing control was added at a fraction of 1% of total purified mRNA. Purified mRNA with Sequin was used to prepare libraries using the KAPA Stranded RNA-seq Library Preparation Kit (Roche, CA, USA) with SeqCap Adapters (Roche CA, USA) as per manufacturer’s instructions. The libraries were sequenced on an Illumina NextSeq 500 System, generating 24.6-48.9 million, 2 x 150bp read-pairs per sample.

#### Nanopore direct-RNA sequencing

Direct RNA sequencing was performed using the ONT Direct RNA Sequencing Kit (SQK-RNA002), as per the manufacturer’s instructions. ∼200 ng of purified mRNA per sample was provided as input and optional first-strand cDNA synthesis was performed using SuperScript III Reverse Transcriptase (Thermo Fisher Scientific). For each sample, ∼30-50 ng of prepped library was loaded onto a single ONT PromethION flow cell and sequenced for a maximum ∼72 hours, generating 2.2-4.6 million native RNA reads per sample.

#### qPCR validation

RNA was reverse-transcribed to complementary DNA using the Applied Biosystem® High Capacity cDNA Reverse Transcription Kit (10X RT Buffer, 25X dNTP mix (100mM), 10X RT Random Primers, Multiscribe® Reverse Transcriptase) on the Applied Biosystem® GeneAmp PCR System 9700 (Thermofisher®, Waltham, MA, USA). qRT-PCR was carried out on the StepOnePlus® PCR machine (Thermofisher®, Waltham, MA, USA) using the following primer assays designed by Integrated DNA Techologies (Skokie, Illinois, USA). Samples were heated to 95°C x 10 min, and then subjected to 40 cycles of amplification by melting at 95°C x 15 s and annealing at 60°C x 1 min. Experimental samples were run in duplicates with 1.33 μL complementary DNA (cDNA) per reaction. To check for amplicon contamination, each run also contained template free controls for each probe used. PCR data were normalized using β-actin and transformed using the ΔΔCt method.

### Quantification and statistical analysis

#### Bioinformatics – gene expression profiling

Reads were trimmed using Trim Galore (Galaxy version 0.6.3) and subsequently aligned to the reference *Mus musculus* genome (GRCm38) using HISAT2 (Galaxy version 2.1.0). Reads were annotated (Gencode v.M25) and counted using featureCounts (Galaxy version 2.0.1). Biostatistics and visualization were run in R (version 3.6.3) with the Rstudio GUI (version 1.2.5033). For each comparison – male: stress(3) v control(3), female:stress(3) v control(3) -genes were filtered to have at least 5 reads in at least 2 samples for each gene and have gene biotype of protein-coding, long non-coding or microRNA. RUVSeq (Risso et al., 2014) and DESeq2 (Love et al,. 2014) were then used to quantify differential gene expression between groups. We set a threshold criteria of a minimum 0.2 expression fold-change and FDR<0.05. Gene ontology and enrichment analysis was performed using EnrichR with a significance threshold set at FDR <0.05 .

#### Bioinformatics – Epitranscriptome profiling

To generate a comprehensive catalogue of high-confidence RNA modification sites present in the amygdala of male and female mice exposed to conditioned fear, we used two recently released software Nanocompore and Xpore on our direct RNA-seq libraries. Nanocompore collects current intensity and dwell time at each position, as the native RNA goes through the pore, and uses those variables to perform pairwise statistical comparisons between control and stress samples. The tool reports positions with a significant statistical difference for each of the individual variables, but also models a logistic regression that takes both current intensity and dwell time into account in search for significant differences. To minimise the false-positive discovery rate, we applied strict filtering to the results, only retaining putative modifications with a significant p-value (alpha=0.01) in all three statistical tests. Xpore, on the other hand, models the current intensity as a mixture of two Gaussian distributions, for modified and unmodified RNA, using prior information to help guide parameter estimation. Then given two conditions, such as control and stress, the tool is able to identify differential modification, but also quantitate them. We only selected putative modifications with significant differences in modification rates (t-test, p-value < 0.01) between the conditions and that were increasingly modified in the stress mice. To further increase the confidence in our dataset, we only retained modifications identified by both Nanocompore and Xpore, and that were at most 10 nt apart from each other and we also collapsed modifications less than 10 nt apart into a single modification site. We compared the coordinates of the modification sites identified using this approach against modification sites previously annotated in RMBase v2.06. We also performed gene set enrichment analysis using EnrichR as described above.

#### Statistical analysis

Cued-fear expression data was analysed using ANOVA with significance threshold set at p<0.05 (∗∗p<0.01 & ∗∗∗p<0.001). For all bioinformatics analyses (see supplemental information), a significance threshold was set at FDR <0.05.

## Supplemental information

Document S1. Figures S1 and Tables S1, S2, S7, and S13

Table S3. Differentially expressed genes in female mice compared with males, control condition, related to Figure 1

Table S4. Differentially expressed genes in female mice compared with males—shock condition, related to Figure 1

Table S5. Differentially expressed genes in male mice, related to Figure 1

Table S6. Enrichment analysis of differentially expressed genes in male mice, related to Figure 1

Table S8. ONT sequencing isoform quantification, related to Figure 1

Table S9. Differentially modified transcripts in male mice, related to Figure 2

Table S10. Differentially modified transcripts in female mice, related to Figure 2

Table S11. Enrichment analysis of differentially modified transcripts in male mice, related to Figure 2

Table S12. Enrichment analysis of differentially modified transcripts in female mice, related to Figure 2

Table S14. Differentially expressed genes that were also differentially modified in male mice, related to Figure 2
