# Supplementary material for: Sex-specific transcriptomic and epitranscriptomic signatures of PTSD-like fear acquisition
Source: iScience. 2022 Aug 2;25(9):104861. doi: 10.1016/j.isci.2022.104861 (PMC9418440; doi:10.1016/j.isci.2022.104861)
Supplement: Document S1. Figures S1 and Tables S1, S2, S7, and S13 [file mmc1.pdf]

iScience, Volume *25*

Supplemental information

Sex-specific transcriptomic and epitranscriptomic signatures of PTSD-like fear acquisition

Andre L.M. Reis, Jillian M. Hammond, Igor Stevanovski, Jonathon C. Arnold, Iain S. McGregor, Ira W. Deveson, and Anand Gururajan

iScience, Volume ■ ■

Supplemental information

Sex-specific transcriptomic and epitranscriptomic signatures of PTSD-like fear acquisition

Andre L.M. Reis, Jillian M. Hammond, Igor Stevanovski, Jonathon C. Arnold, Iain S. McGregor, Ira W. Deveson, and Anand Gururajan

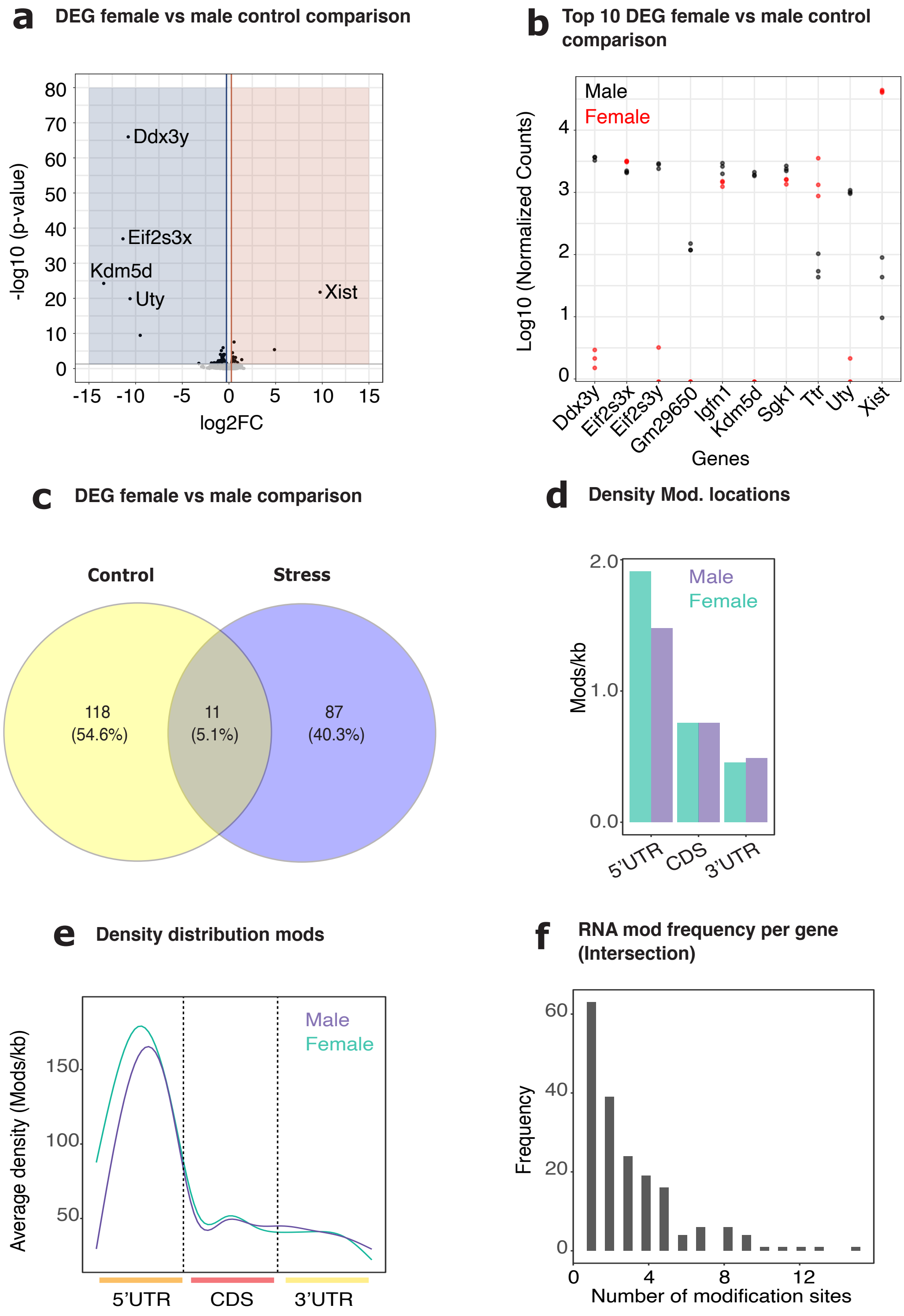

Figure S1. Male versus female comparison and RNA modification features, Related to Figures 1 and 2 – (**a**) Volcano plot showing differential gene expression comparison between male and female control samples; (**b**) Normalized counts of top 10 differentially expressed genes between male and female control samples; (**c**) Intersection between male and female differentially expressed genes in control and stress conditions; (**d**) Density of modification distribution; (**e**) Relative location of RNA modifications within transcripts; (**f**) Number of modifications per gene, for genes with modifications in both male and female mice.

Table S1. Sample information and RIN Analysis, Related to Figure 1.

	#	Group	ID	Conc (ng/ul)	260/280	260/230	Factor	Yield (ug)	Pool	Pool total	RINe
**Male**	1	Control	C1	69.1	2.08	2.08	40	5.53	CM1		
**Male**	2	Control	C2	87.8	2.02	1.70	40	7.02	CM1		
**Male**	3	Control	C3	128.5	2.08	2.18	40	10.28	CM1		
**Male**	4	Control	C4	144.7	2.07	1.50	40	11.58	CM2		
**Male**	5	Control	C5	116.3	2.07	2.10	40	9.30	CM2		
**Male**	6	Control	C6	71.4	2.01	1.71	40	5.71	CM1	28.54	7.00
**Male**	7	Control	C7	100.2	2.06	1.67	40	8.02	CM2	28.90	7.20
**Male**	8	Control	C8	113.7	2.06	1.79	40	9.10	CM3		
**Male**	9	Control	C10	155.1	2.08	1.90	40	12.41	CM3		
**Male**	10	Control	C12	91.1	2.07	0.51	40	7.29	CM3	28.79	7.30
**Male**	11	Shock	S1	89.7	2.04	0.80	40	7.18	SM1	22.13	7.80
**Male**	12	Shock	S2	47.3	2.03	1.96	40	3.78	SM1		
**Male**	13	Shock	S3	57.9	2.03	1.58	40	4.63	SM1		
**Male**	14	Shock	S4	81.7	2.03	1.86	40	6.54	SM1		
**Male**	15	Shock	S5	59.8	2.03	2.02	40	4.78	SM2	21.72	7.60
**Male**	16	Shock	S6	93.7	1.93	1.14	40	7.50	SM2		
**Male**	17	Shock	S7	29.3	1.97	1.63	40	2.34	SM2		
**Male**	18	Shock	S8	88.7	1.72	0.91	40	7.10	SM2		
**Male**	19	Shock	S9	76.9	2.02	1.68	40	6.15	SM3	20.82	7.80
**Male**	20	Shock	S10	106.0	2.03	2.04	40	8.48	SM3		
**Male**	21	Shock	S11	51.0	2.03	0.79	40	4.08	SM3		
**Male**	22	Shock	S12	26.3	2.03	1.57	40	2.10	SM3		
**Female**	1	Control	C1	67.5	2.05	1.22	40	5.40	CF1	19.28	6.90
**Female**	2	Control	C2	48.3	1.88	1.21	40	3.86	CF1		
**Female**	3	Control	C3	57.7	2.05	0.57	40	4.62	CF1		
**Female**	4	Control	C4	67.5	2.05	0.71	40	5.40	CF1		
**Female**	5	Control	C5	76.2	2.04	0.47	40	6.10	CF2	20.28	7.60
**Female**	6	Control	C6	44.4	2.05	1.50	40	3.55	CF2		
**Female**	7	Control	C7	50.8	2.04	2.09	40	4.06	CF2		
**Female**	8	Control	C8	51.3	2.04	1.94	40	4.10	CF3	21.89	7.60
**Female**	9	Control	C9	82.1	2.04	1.85	40	6.57	CF2		
**Female**	10	Control	C10	68.1	2.02	1.45	40	5.45	CF3		
**Female**	11	Control	C11	105.5	2.04	1.95	40	8.44	CF3		
**Female**	12	Control	C12	48.7	2.04	2.02	40	3.90	CF3		
**Female**	13	Shock	S1	46.9	1.97	1.99	40	3.75	SF1	21.26	7.70
**Female**	14	Shock	S2	25.8	1.96	1.48	40	2.06	SF1		
**Female**	15	Shock	S3	30.1	1.98	1.34	40	2.41	SF2	21.10	7.40
**Female**	16	Shock	S4	40.1	1.97	1.48	40	3.21	SF2		
**Female**	17	Shock	S5	74.7	1.97	1.68	40	5.98	SF3	23.01	7.60
**Female**	18	Shock	S6	67.6	1.92	0.88	40	5.41	SF2		
**Female**	19	Shock	S7	81.5	2.01	2.00	40	6.52	SF3		
**Female**	20	Shock	S8	64.8	2.00	0.58	40	5.18	SF3		
**Female**	21	Shock	S9	126.0	2.03	2.07	40	10.08	SF2		
**Female**	22	Shock	S10	100.7	1.80	1.07	40	8.06	SF1		
**Female**	23	Shock	S11	66.6	2.01	1.80	40	5.33	SF3		
**Female**	24	Shock	S12	92.4	2.03	2.13	40	7.39	SF1		

Table S2. Illumina sequencing metadata, Related to Figure 1.

Gender	Group	ID	Read pairs	Alignment (%)	Reads assigned	Reads unassigned (amiguity)	Reads unassigned (multimapping)	Reads unassigned (no feat.)	Reads unassigned (unmapped)
Male	Control	C1	41487699	85.23	55949127	2289614	11300450	11229093	8692278
Male	Control	C2	39393294	83.9	50565002	2057963	10710257	12458723	9252499
Male	Control	C3	37502359	84.61	49398166	2032017	10339008	10881070	8267739
Male	Stress	S1	45332871	84.65	60273069	2448821	11706633	12910950	10078806
Male	Stress	S2	34861558	84.87	34781588	1400166	5983099	8170388	5713196
Male	Stress	S3	26325794	84.78	45521175	1830299	8861354	11056583	7603869
Female	Control	C1	48915566	85.09	33139753	1332423	6127105	7300141	4959895
Female	Control	C2	35016314	84.31	45066193	1828363	9001603	11505386	7935474
Female	Control	C3	24663292	85.77	65078425	2664988	13685579	13970645	10401297
Female	Stress	S1	45325334	83.98	51215833	2088257	10379916	11878146	8932234
Female	Stress	S2	37656809	85.22	49906147	2038034	9862821	11206717	8050096
Female	Stress	S3	39195830	84.22	58938048	2404931	12995183	13721877	10294202

Table S7. ONT sequencing metadata, Related to Figure 1.

Group	Gender	ID	Passed reads	Passed yield	Average read length	Maximum read length
control	female	C1	3,513,548	5,231,763,871	1,489	18,219
control	female	C2	4,395,482	6,539,080,227	1,487.70	28,726
control	female	C3	4,619,328	5,197,619,941	1,125.20	15,692
control	male	C1	4,360,179	6,557,971,239	1,504.10	19,488
control	male	C2	4,287,537	5,864,457,464	1,367.80	18,344
control	male	C3	3,206,257	4,667,269,577	1,455.70	19,834
stress	female	S1	3,748,572	5,782,887,483	1,542.70	26,121
stress	female	S2	3,470,134	5,120,664,648	1,475.60	18,295
stress	female	S3	4,116,830	5,945,668,648	1,444.20	21,065
stress	male	S1	3,569,172	5,672,176,624	1,589.20	22,783
stress	male	S2	4,549,131	6,751,211,796	1,484.10	22,839
stress	male	S3	2,241,571	3,051,599,156	1,361.40	18,758

Table S13. Enrichment analysis of common differentially modified transcripts in male and female mice, Related to Figure 2.

Term	Overlap	P.value	Adjusted P.value	Old P.value	Old Adjusted P.value	Odds.Ratio	Combined.Score	Genes
chemical synaptic transmission (GO:0007268)	0.0458	1.12E-06	0.001755834	0	0	5.441780822	74.58260604	GRIA2;SNAP25;NAPB;GABBR2;SLC12A5;STXBP1;SLC1A2;NSG1;SLC6A1;CPLX1;PRKAR1B;DNAJC5;PRKACB;SNCA
cotranslational protein targeting to membrane (GO:0006613)	0.0851	2.71E-06	0.002118703	0	0	10.30990332	132.1646793	RPL7A;RPL32;RPLP0;RPS6;SRP14;SEC62;RPL6;RPL7

neurotransmitter transport (GO:0006836)	0.0959	5.22E-06	0.002118703	0	0	11.70103267	142.3271737	SNAP25;DNAJC5;STXBP1;SLC1A2;ATP1A2;GLUL;CPLX1
protein targeting to ER (GO:0045047)	0.0777	5.39E-06	0.002118703	0	0	9.3289178	113.1728031	RPL7A;RPL32;RPS6;RPLP0;SRP14;SEC62;RPL6;RPL7
SRP-dependent cotranslational protein targeting to membrane (GO:0006614)	0.0778	2.09E-05	0.006589679	0	0	9.296425927	100.1555344	RPL7A;RPL32;RPLP0;RPS6;SRP14;RPL6;RPL7
signal release from synapse (GO:0099643)	0.1163	4.76E-05	0.008391074	0	0	14.37627217	143.0705157	SNAP25;STXBP1;DNAJC5;CPLX1;SNCA
negative regulation of proteolysis (GO:0045861)	0.1163	4.76E-05	0.008391074	0	0	14.37627217	143.0705157	CST3;PRNP;TMEM59;SERPINE2;CAMK2N1
aerobic electron transport chain (GO:0019646)	0.0857	4.78E-05	0.008391074	0	0	10.28645833	102.3444907	NDUFB6;NDUFA4;NDUFS3;NDUFC1;SDHC;COX5B
mitochondrial ATP synthesis coupled electron transport (GO:0042775)	0.0845	5.18E-05	0.008391074	0	0	10.12769231	99.9484642	NDUFB6;NDUFA4;NDUFS3;NDUFC1;SDHC;COX5B
neurotransmitter secretion (GO:0007269)	0.1136	5.33E-05	0.008391074	0	0	14.00694149	137.8106767	SNAP25;STXBP1;DNAJC5;CPLX1;SNCA
positive regulation of ATP biosynthetic process	0.3333	6.38E-05	0.008720588	0	0	54.12021858	522.7895301	PINK1;VCP;ENO1

(GO:2001171)								
potassium ion homeostasis (GO:0055075)	0.1667	6.65E-05	0.008720588	0	0	21.75164835	209.2052201	SLC12A5;ATP1A2;ATP1A1;ATP1B1
inorganic cation import across plasma membrane (GO:0098659)	0.0779	8.18E-05	0.009901875	0	0	9.269014085	87.22903874	SLC12A5;SLC39A10;ATP1A2;ATP1A1;SLC6A1;ATP1B1
